# Multiple Regulatory Mechanisms Control B-1 B Cell Activation

**DOI:** 10.3389/fimmu.2012.00372

**Published:** 2012-12-17

**Authors:** Vishal J. Sindhava, Subbarao Bondada

**Affiliations:** ^1^Department of Microbiology, Immunology and Molecular Genetics, University of Kentucky College of MedicineLexington, KY, USA; ^2^Markey Cancer Center, University of Kentucky College of MedicineLexington, KY, USA

**Keywords:** B lymphocyte, B-1 cell, B cell receptor, Toll-like receptor, CD5, SHP-1, CD19, IL-10

## Abstract

B-1 cells constitute a unique subset of B cells identified in several species including mice and humans. B-1 cells are further subdivided into B-1a and B-1b subsets as the former but not the later express CD5. The B-1a subset contributes to innate type of immune responses while the B-1b B cell subset contributes to adaptive responses. B-1 cell responses to B cell receptor (BCR) as well as Toll-like receptor (TLR) ligation are tightly regulated due to the cross-reactivity of antigen specific receptors on B-1 cells to self-antigens. B-1 cells are elevated in several autoimmune diseases. CD5 plays a major role in down regulation of BCR responses in the B-1a cell subset. Reduced amplification of BCR induced signals via CD19 and autoregulation of BCR and TLR responses by B-1 cell produced IL-10 appear to have a role in regulation of both B-1a and B-1b B cell responses. Siglec G receptors and Lyn kinase also regulate B-1 cell responses but their differential role in the two B-1 cell subsets is unknown.

## Introduction

B cells are heterogeneous in their surface phenotypes, anatomical localization, capacity for self-renewal, and functional properties. The two major subsets of the B cells are B-2 and B-1 B cells, which were initially defined by differential expression of a classical T cell specific differentiation antigen, CD5 (expressed on B-1 cells). The B cell receptors (BCRs) on B-1 cells exhibit polyreactivity enabling them to respond to conserved epitopes on microbes, but that also leads to cross-reactivity with self-antigens. In this review we summarize the mechanisms involved in regulation of BCR and Toll-like receptor (TLR) mediated B-1 cell activation.

## B-2 Cells

B-2 cells are produced in bone marrow from hematopoietic stem cells and migrate to secondary lymphoid organs as immature B cells. Transitional B cells are the most immature B cells in the spleen and are the crucial link between bone marrow immature and peripheral mature B cells (Chung et al., 2003). These transitional B cells are called the T1 subset when they first emerge from bone marrow, which mature into T2 subset mainly in the spleen. The T1 subset is a stage of negative selection against self-reactive B cells that have escaped central tolerance mechanisms in the bone marrow (Cancro et al., 1998). The T2 B cells further mature and differentiate into follicular and marginal zone B cells (Miller et al., 2006). B cell-activating factor, BAFF (a.k.a. B Lymphocyte Stimulator, BLyS) is essential for the survival of mature follicular and marginal zone B cells. In both BAFF receptor mutant and BAFF knockout mice, most transitional B cells fail to differentiate into follicular B cells, and the few follicular B cells formed have a short lifespan (Lentz et al., 1996; Gross et al., 2001; Schiemann et al., 2001; Gavin et al., 2005). BAFF signaling mainly promotes B cell survival, as enforced expression of anti-apoptotic factors Bcl-2 or Bcl-xL restored splenic B lymphocyte development in BAFF-R mutant mice (Amanna et al., 2003; Tardivel et al., 2004). Upon exposure to antigen, follicular B cells undergo clonal expansion, Ig class switching, and differentiation into plasma and memory B cells (McHeyzer-Williams and McHeyzer-Williams, 2005). The transitional, follicular, and marginal zone B cells comprise the B-2 subset.

## B-1 Cells

B-1 cells were first identified on the basis of expression of CD5, a pan T cell marker, on a subset of B cells (Manohar et al., 1982; Hayakawa et al., 1983). B-1 cells are mainly present in peritoneal cavities, pleural cavities, and various parts of intestine and constitute only a small fraction of B cells in the spleen (Kroese et al., 1992). B-1 cell origin and development occurs primarily during fetal and perinatal life. There have been two different models proposed for the origin of the B-1 cells, lineage model, and selection model. The lineage model supports the existence of distinct progenitors for B-1 and B-2 cells (Hardy and Hayakawa, 1991; Kantor et al., 1992; Herzenberg and Tung, 2006). Thus transfer of fetal liver cells reconstituted both B-1 and B-2 cell populations, whereas adult bone marrow transfer reconstituted conventional B (B-2) cells but not B-1 cells. In contrast, the selection model proposes a common progenitor for both B-1 and B-2 cells and antigen selection (antigenic stimuli) determines the development of B cells with B-1 or B-2 characteristics (Lam and Rajewsky, 1999; Berland and Wortis, 2002). Studies showing that CD5 expression can be induced by BCR signaling in the presence of certain cytokines and BCRs specific to some antigens support antigen selection models. Lam and Rajewsky (1999) showed that co-expression of non B-1 specific BCR (V_H_B-1-8 or V_H_glD42) along with B-1 specific BCR (V_H_12) on B cells leads to development of B-2 but not B-1 cells. They proposed that expression of non B-1 specific BCR dilutes out V_H_12-containing BCR complexes on the cell surface and presumably acts in a “dominant-negative” manner and may not provide sufficient signals for the development of B-1 phenotype. This can perhaps best be explained by postulating that signaling via a BCR of a certain specificity, expressed at the cell surface at high density, is required to drive the differentiation of B cells into the B-1 subset (Lam and Rajewsky, 1999). Both the models are well supported by evidence and are the subject of considerable debate. However, a recent study from Dorshkind and colleagues identified specific B-1 cell restricted progenitors (Lin^−^CD45R^lo-neg^CD19^+^ cells) in bone marrow, which preferentially reconstituted functional B-1 B cells, but not B-2 B cells, *in vivo*, providing strong support to the lineage model (Montecino-Rodriguez et al., 2006). Moreover, using single hematopoietic stem cells for reconstitution Ghosn et al. (2012) demonstrated that B-1a cell lineage derives from a precursor that is distinct from other B cell lineages.

## B-1 Cell Subsets

Along with the presence of CD5 on the surface, B-1 cells are further differentiated from B-2 cells by surface expression of CD11b (Mac-1), high levels of IgM, and low levels of IgD (Tung et al., 2004). B-1 cells are subdivided into three different subsets, B-1a, B-1b, and B-1c, on the basis of CD5 and CD11b expression. B-1a cells are CD11b^+^CD5^+^, B-1b cells are CD11b^+^CD5^−^, and the newly described rare subpopulation of B-1c cells is CD11b^−^CD5^+^ (Tung et al., 2004; Hastings et al., 2006). B-1a cells have a role in innate immunity via their contribution to natural antibodies, while B-1b cells are critical in development of IgM memory cells (Berland and Wortis, 2002). The functional properties of B-1c are essentially similar to those of B-1a and B-1b B cells (Hastings et al., 2006). The unique markers for B-1 cells in the human were unclear since CD5 was expressed by activated human B cells. More recently, Griffin et al. (2011) have characterized human B-1 cell surface phenotype and function, which resembled the properties of murine B-1 cells very closely (Griffin et al., 2011; Griffin and Rothstein, 2012).

B-1 cells are important in protection against certain bacterial infections such as *S. pneumoniae*, *Borrelia hermsii* as well as in the early IgM response against viruses such as influenza (Baumgarth et al., 2000; Alugupalli et al., 2003; Haas et al., 2005). Despite their role in protection against infection, B-1 cell antibodies have been found to be poly-reactive and as such are reactive to self-antigens such as those on red blood cells, thy 1.2, single stranded DNA (Berland and Wortis, 2002). Moreover, B-1 cells have been found to be elevated in autoimmune diseases both in mouse and human. In mouse models, elimination of B-1 cells by genetic deficiency reduced autoimmunity (Duan and Morel, 2006).

### BCR signaling in B-1 cells

B cell receptor signaling plays a critical role in B-1 cell development, survival, or expansion. Transgenic mice or mice with mutations that disrupt BCR signaling have a decrease in B-1 cell numbers, and mutations that enhance BCR signaling result in increased B-1 cell compartment (Berland and Wortis, 2002). However, the cross-reactivity of B-1 BCRs with self-antigens raised the question of how B-1 cells are prevented from activation via self-antigens in the absence of overt infection. Studies of BCR signaling have demonstrated distinct differences between B-1 and B-2 cells. Engagement of BCR on B-2 cells leads to robust intracellular calcium mobilization and proliferation, while in B-1 cells, BCR ligation induces modest calcium mobilization, little or no proliferation, and increased apoptosis (Murakami et al., 1992; Morris and Rothstein, 1993; Bikah et al., 1996; Sen et al., 1999). Here we summarized the key molecules that negatively regulate BCR and TLR signaling in B-1 cells and have a role in B-1 cell hypo-responsiveness to BCR ligation.

#### Negative regulatory role of CD5 in B-1a cells

CD5 is a 67-kDa monomeric type 1 transmembrane glycoprotein, historically also known as Lyt-1 or Ly-1. Extracellular domains of CD5 are characterized by the presence of the highly conserved scavenger receptor cysteine-rich domain. CD5 expression was first identified on T cells (Boyse et al., 1968) and subsequently shown to be expressed on B cells (Manohar et al., 1982; Okumura et al., 1982; Hardy et al., 1983; Hayakawa et al., 1983). CD5^+^ B cells, later termed B-1a cells, have unique function of “spontaneous” IgM secretion that contributes to natural antibodies (Hayakawa et al., 1983). Also, B-1 cells have a limited BCR repertoire with dominant cross-reactivity to self-antigens, but expansion of these poly-reactive B-1 cells is limited (Berland and Wortis, 2002). This limited expansion of self-reactive B-1 cells may be in part due to the presence of various mechanisms that negatively regulate BCR signaling.

Various studies identified CD5 as one of the negative regulators of BCR signaling, similar to its ability to inhibit T cell function (Tarakhovsky et al., 1995). In B cells CD5 associates with mIgM upon BCR stimulation (Lankester et al., 1994). However, CD5 is shown to be constitutively associated with mIgM in peritoneal B-1 cells (Sen et al., 1999). Bikah et al. (1996) demonstrated for the first time that CD5 negatively regulates BCR signaling in peritoneal B-1 cells. B-1 cells from both wild type (WT) and CD5 KO mice proliferated comparably in response to anti-CD40 and LPS. However, only CD5 KO B-1 cells, but not WT B-1 cells, proliferated to anti-IgM stimulation. This involved sustained calcium mobilization and increased nuclear localization of NF-κB following BCR ligation in CD5 KO compared to WT peritoneal B-1 cells. Additionally, blocking of CD5 association with mIgM rescued the proliferative defect of B-1 cells upon BCR ligation (Bikah et al., 1996). Using a novel fusion protein containing the extracellular and transmembrane domains of FcγRIIB and the cytoplasmic region of CD5 Gary-Gouy et al. (2000, 2002) showed that co-cross-linking of BCR with the chimeric protein induced tyrosine phosphorylation in CD5 cytoplasmic tail along with rapid inhibition of BCR induced calcium transients and extracellular regulated kinase-2 (ERK2) activation. Subsequent they showed that Y429, residue outside the putative immune receptor tyrosine based inhibitory motif (ITIM) of CD5 cytoplasmic domain is responsible for the inhibition of BCR induced calcium response, Akt relocalization (Gary-Gouy et al., 2002), activation of the Ras/ERK2 pathway as well as IL-2 production (Gary-Gouy et al., 2002).

Using the well-known transgenic mouse model of anti-hen egg lysozyme (HEL) crossed to transgenic mice expressing soluble HEL (Goodnow et al., 1988), Hippen and Behrens showed that anergic B cells expressed significant surface levels of CD5, though lower than those expressed on typical B-1a cells. This suggested that a low level of CD5 induction on B cells upon stimulation through auto-antigen might be sufficient to induce an anergic state in self-reactive B cells and thus limiting production of autoantibodies. Consistent with such a concept, CD5 KO mice, but not CD5^+^ mice, that are transgenic for both HEL specific BCR and soluble lysozyme produced antibody to the self-antigen, HEL (Hippen et al., 2000). Also, the CD5 KO HEL specific B cells that are no longer anergic showed enhanced proliferative responses and calcium mobilization upon BCR ligation. Together, data from Bikah et al. (1996) and by Hippen et al. (2000) suggested that CD5 negatively regulates BCR signaling and limits self-reactive B cell responses. Similar to anergic B cells in the transgenic model, constitutive expression of CD5 on B-1a cells might also play a role in limiting the expansion of auto-reactive B-1a cells.

#### Lyn, SHP-1, CD22 and Siglec G

B cell receptor signal transduction occurs via activation of several protein tyrosine kinases (PTK), including members of the Src family kinases (SFKs; Cambier et al., 1994). In addition to taking part in activation of the BCR signaling, Lyn, an SFK, negatively regulates BCR signaling by phosphorylating the ITIM motifs in B cell co-receptors (DeFranco et al., 1998). Phosphorylation of ITIM motif with Lyn induces the recruitment of negative molecular switches, like protein tyrosine phosphatases (PTP; Thomas, 1995). Dasu et al. (2009) also made the surprising observation that B-1 cells have a constitutively active Lyn. Lyn appears to have dual roles in B-1 cells such that high doses provide negative signals, whereas small amounts of Lyn were essential for B-1 cell activation as demonstrated by rescue of both proliferation and calcium responses at low doses of Lyn kinase inhibitors (Dasu et al., 2009).

Several PTPs like, tyrosine-protein phosphatase non-receptor type 6 (SHP-1/PTPN6), SHP-2, and inositol polyphosphate 5′ phosphatase are involved in the inhibition of BCR signaling (Thomas, 1995; Long, 1999). In B cells, SHP-1 associates with inhibitory receptors like FcR, CD22, and paired Ig-like receptor, PIR-B (Doody et al., 1995; Pani et al., 1995; Long, 1999). Motheaten and the viable motheaten mice with mutations in the SHP-1 enzyme exhibit autoimmunity and accelerated mortality due to the presence of hyper-responsive B-1 cells (Cyster and Goodnow, 1995). Moreover, B cell specific deletion of SHP-1 leads to an expansion of B-1 cells, rescue of BCR induced calcium response and autoimmunity characterized by anti-DNA antibodies (Pao et al., 2007).

Cytoplasmic domain of CD5 contains an amino acid sequence (LAY^378^KKL), with excellent homology to ITIMs of inhibitory receptors (Perez-Villar et al., 1999) and thus can interact with SHP-1. There is a constitutive association of the BCR with SHP-1 in both B-1 and B-2 cells (Sen et al., 1999). Upon BCR ligation association of SHP-1 is decreased in splenic B-2 cells, but not in peritoneal B-1 cells. The persistent BCR-SHP-1 association is mediated by CD5 in peritoneal B-1 cells and is lost in CD5 KO peritoneal B-1 cells or in wild type B-1 cells when CD5 is sequestered away from BCR (Sen et al., 1999). These data suggest that CD5 may negatively regulate BCR-mediated growth signals by recruiting SHP-1 into the BCR complex in B-1 cells.

The B cell surface molecules CD22 and CD72 can also associate with SHP-1, but were not found to have a critical role in the anergic state of B-1 cells (Ochi and Watanabe, 2000; Lajaunias et al., 2002). However, Siglec G, another member of the CD22 family of sialic acid binding proteins, is important for both B-1 cell development and for regulating BCR induced calcium responses, but not BCR induced B-1 cell proliferation (Hoffmann et al., 2007). Interestingly, CD22^−/−^Siglec G^−/−^ double knockout mice had even greater elevation of BCR induced calcium responses in B-1 cells suggesting a role for both molecules in regulating BCR responses in B-1 cells (Jellusova et al., 2010a,b). These double knockouts develop anti-nuclear antibodies and glomerular nephritis (Jellusova et al., 2010b).

#### CD19

The inability of B-1a cells to respond well to BCR signaling with a role for negative regulation by CD5 and SHP-1 is described above. B-1b B cells, which are CD5 negative by definition, are equally defective in proliferation induced by BCR cross-linking raising the possibility that additional mechanisms may exist to downregulate BCR responses in B-1 cells (Sen et al., 1999). CD19, a co-receptor expressed by all B cell subsets, serves as a positive regulator of BCR signaling and is critical for B cell development and activation (Cambier et al., 1994; Tedder et al., 1994). CD19 is shown to be involved in the development and self-renewal of B-1 cells (Krop et al., 1996; Sato et al., 1996). Signals from CD19 and BCR act in synergy to induce robust calcium mobilization in splenic B-2 cells (Carter et al., 1991; Fujimoto et al., 2001). Using this criterion, Sen et al. (2002) found that both B-1a and B-1b B cells are equally hypo-responsive to synergistic calcium mobilization obtained by co-cross-linking BCR and CD19 compared to B-2 cells. CD19 cross-linking amplifies BCR signaling in part by recruitment of Vav, a guanine nucleotide exchange factor for the Rho, Rac, and Cdc42 family of small GTPases (O’Rourke et al., 1998). Vav binds to phosphorylated tyrosine-391 of CD19 to mediate a sustained increase in intracellular Ca2^+^ concentration and activation of the mitogen-activated protein kinase, JNK (O’Rourke et al., 1998). Association of CD19 with Vav is reduced in B-1 cells (Sen et al., 2002). Similarly BCR dependent proliferation as well as CD19 mediated augmentation of BCR induced calcium elevation were deficient in B-1a and B-1b B cell subsets from both spleen and peritoneal cavity (Dasu et al., 2009). The defects in calcium response were mainly in the mobilization of extracellular calcium by both B-1a and B-1b B cells stimulated via CD19 and BCR.

### IL-10 and TLR signaling in B-1 cells

Toll-like receptors are pattern recognition receptors that recognize pathogen-associated molecular patterns. So far eleven functional TLRs (TLR8 and TLR10 genes do not encode functional proteins) have been described in the mouse (Browne, 2012). Several B cell subsets express TLRs and can be activated via TLR ligands that result in robust proliferation and antibody secretion, even in the absence of dendritic cell or T cell help (Genestier et al., 2007; Gururajan et al., 2007). TLR mediated signals synergize with self-antigen-mediated BCR signals to stimulate activation of self-reactive B cells (Leadbetter et al., 2002), and B cell activation was severely reduced when the mice were deficient in TLR signaling (Leadbetter et al., 2002; Browne, 2012). B-1 cells express various TLRs (TLR1, 2, 3, 4, 7, 8, and 9; Gururajan et al., 2007). B-1 cells are more prone to terminal plasma cell differentiation than B-2 cells upon TLR stimulation (Genestier et al., 2007). B-1 cell activation and B-1 cell mediated auto-antibody production are augmented upon stimulation via TLR4 or TLR9 (Murakami et al., 1994; Kubo et al., 2009). Also, TLR signaling in B-1 cells plays an important role in the clearance of various pathogens such as influenza virus, pneumococcus, and *Borrelia* spp. (Baumgarth et al., 2000; Alugupalli et al., 2003; Haas et al., 2005). Sindhava et al. (2010) showed that peritoneal B-1 cells are hypo-responsive to various TLR ligands compared to B-2 cells.

Peritoneal B-1 cells were the first B cell subset to be reported as being high IL-10 producers (O’Garra et al., 1992). Recently human CD11b^+^ B-1 cells were also found to spontaneously secrete IL-10 (Griffin and Rothstein, 2012). IL-10 is a potent regulator of immune function through its ability to inhibit antigen presentation, pro-inflammatory cytokine production, T cell proliferation, and acts as a key effector molecule for certain types of regulatory T and B cells. As such, IL-10 production by B-1 cells raises the possibility that B-1 cells may regulate their own function and/or the function of other immunocompetent cells. Sindhava et al. (2010) showed that among different B cell subsets from spleen and peritoneum, B-1 cells from the peritoneum are the major IL-10 producers. Peritoneal B-1a cells produced highest level of IL-10 followed by B-1b cells, whereas IL-10 production by peritoneal B-2 cells was minimal both constitutively and upon TLR stimulation (Sindhava et al., 2010). The high levels of IL-10 produced by peritoneal B-1 cells regulated the TLR as well as BCR (Sindhava et al., unpublished results) induced B-1 cell proliferation in an autocrine fashion, as B-1 cells from IL-10 knockout mice proliferated significantly more than WT B-1 cells both *in vitro* and *in vivo*. IL-10 regulated B-1 cell response to TLR by inhibiting classical NF-κB signaling. Furthermore, IL-10 produced by peritoneal B-1 cells limits the clearance of *B. hermsii* infection (Sindhava et al., 2010). Thus, similar to BCR signaling, TLR signaling is also regulated in peritoneal B-1 cells, which might prevent excessive activation of self-reactive B cells via TLR stimulation.

## Concluding Remarks

**Figure 1:**
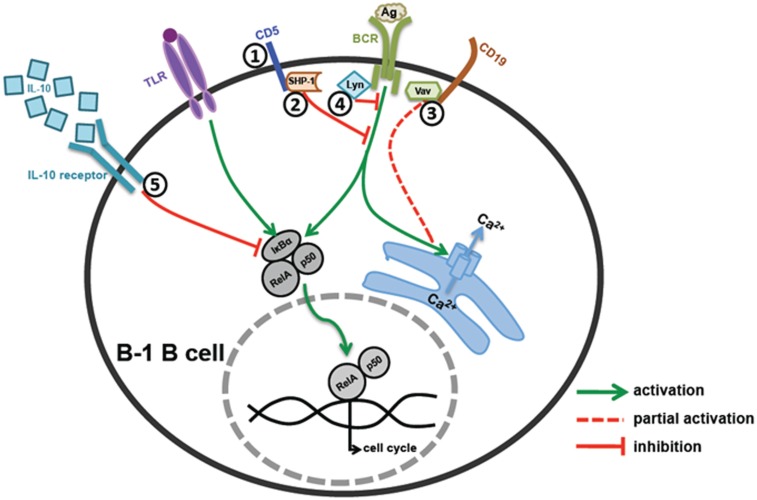
**Regulation of B-1 B cell activation**. (1 and 2) CD5 mediated regulation – CD5 acts as an anchor for SHP-1 recruitment on cell surface near BCR signaling complex, which in turn inhibits BCR signaling. (3) CD19 mediated regulation – B-1 cells have defective Vav recruitment to CD19 leading to reduced Ca2^+^ mobilization and cell activation upon BCR co-stimulation. (4) Src family kinase mediated regulation – Src family kinase, Lyn, plays an essential role in phosphorylation of CD5 and subsequent recruitment of SHP-1 on CD5. (5) IL-10 mediated regulation – B-1 cells make high levels of IL-10 upon TLR and BCR stimulation, which work in an autocrine manner and inhibit B-1 cell responses by blocking degradation of IκBα and RelA translocation to the nucleus.

B-1 cells express poly-reactive BCRs with cross-reactivity to self-antigens. Accidental activation by self-antigens is prevented by multiple mechanisms that keep B-1 cells in anergic state. Lyn, a major negative regulator of BCR signaling phosphorylates ITIMs on inhibitory receptors (CD5, Siglecs, etc.) leading to recruitment of PTPs, which antagonize the BCR mediated activation of PTKs. Anatomical location of B-1 cells makes them prone to activation through microbial TLR ligands that might result in auto-antibody production. IL-10 mediated autoregulation plays a key role in controlling expansion of self-reactive B-1 cells. However, signals from CD40 and high dose TLR ligands can overcome the anergic state of B-1 cells enabling their activation during infection. Defects in the negative regulatory mechanisms may account for elevation of B-1 cells and autoantibodies in lupus like autoimmune diseases. Various molecules that negatively regulate B-1 cell activation are summarized in Figure 1.

## Conflict of Interest Statement

The authors declare that the research was conducted in the absence of any commercial or financial relationships that could be construed as a potential conflict of interest.
